# Living in human-modified landscapes narrows the dietary niche of a specialised mammalian scavenger

**DOI:** 10.1038/s41598-023-30490-6

**Published:** 2023-03-03

**Authors:** Anna C. Lewis, Channing Hughes, Tracey L. Rogers

**Affiliations:** 1 Evolution and Ecology Research Centre, School of Biological, Earth and Environmental Sciences, University of New South Wales, Sydney, NSW Australia; 2 Centre for Marine Science and Innovation, School of Biological, Earth and Environmental Sciences, University of New South Wales, Sydney, NSW Australia; 3 School of Life and Environmental Sciences, The University of Sydney, Sydney, NSW Australia; 4 The Carnivore Conservancy, Ulverstone, TAS Australia

**Keywords:** Ecology, Stable isotope analysis

## Abstract

Anthropogenic impacts on carnivores can be complex, posing numerous threats to many species, yet also benefits to those able to exploit certain resources. This balancing act is particularly precarious for those adapters that exploit dietary resources provided by humans, but still require other resources only available in native habitat. Here we measure the dietary niche of one such species, the Tasmanian devil (*Sarcophilus harrisii*), a specialised mammalian scavenger, across an anthropogenic habitat gradient stretching from cleared pasture to undisturbed rainforest. Populations inhabiting areas of greater disturbance showed restricted dietary niches, suggesting that all individuals fed on similar food items, even within regenerated native forest. Populations in undisturbed rainforest habitats had comparatively broad diets and showed evidence of niche partitioning by body size, which may reduce intraspecific competition. Despite the potential benefits of reliable access to high-quality food items in anthropogenically-modified habitats, the constrained niches we observed may be harmful, indicating altered behaviours and potentially increasing the rate of fights between individuals over food. This is of particular concern for a species at risk of extinction due to a deadly cancer primarily transmitted through aggressive interactions. The lack of diversity in devil diets within regenerated native forest compared to those in old-growth rainforest also indicates the conservation value of the latter for both the devil and the species which they consume.

## Introduction

Anthropogenic influence has resulted in a complex blend of harmful and beneficial outcomes for carnivores. While the extinction risk of many species is undoubtedly linked with their exposure to human activity^1^, not all will respond to threats such as habitat loss or fragmentation, the construction of artificial structures, or direct persecution in exactly the same way^2–8^. To successfully inhabit highly disturbed landscapes, it is predicted that carnivores should be small resource generalists^2^ with a high degree of behavioural plasticity^5^. For these individuals that can avoid, tolerate, or exploit human interactions, certain benefits then outweigh the risks. One such benefit is the increased availability of food items from anthropogenic sources, either through deliberate feeding^9^, from waste^10^, or by provision of carcasses from hunting or as roadkill^4,11–13^. Additionally, humans can supplement carnivore diets indirectly by removing potential competitors and artificially inflating prey density^4,14,15^. This supplementary feeding allows individuals to reduce time and energy spent foraging and exploit a greater access to reliable, high quality food sources, manifesting in advantages such as increased body mass^16–18^ and population density^19–21^.

However, these benefits to certain species can potentially mask hidden consequences. Supplementary feeding, whether intentional or not, has the potential to increase exposure to pathogens and poisons^22–25^, alter foraging behaviour and habitat use^15,20,26–31^, disrupt ecosystem dynamics with competitors, predators, and prey^32–34^, and favour certain individuals or species over others^12,35,36^. On top of this, human disturbance and food supplementation often collapses niche partitioning, leading to reduced diversity in both diet and feeding behaviours^37–39^. Primarily, research that tests the influence of anthropogenic impact on carnivore diet has focused on an urban–rural gradient. This not only may bias research towards those particularly successful species described by Blair as ‘urban exploiters’ rather than ‘adapters’ to human-modified landscapes^40^, but also potentially overlooks the impact of humans on diet in sparsely populated compared to completely undisturbed regions (with some exceptions^17,38,41^). These ‘adapters’ are typically native species that utilise human food or structures yet still require certain resources that are lost from cities or large towns^42^. As such the relationship between these species and humans becomes an exceptionally complicated balancing act.

The Tasmanian devil is one such species maintaining a complex relationship with human society. Although initially considered common by early Europeans, they suffered a population decline—at least in settled regions—in the late 19th and early twentieth century, potentially due to a combination of extensive land clearing, localised persecution by landowners, and disease^43^. As a species, however, they have proved highly adaptable to moderate anthropogenic impacts, with their presence increasing in rural regions of Tasmania from the 1960s^43^. That is until the emergence of the transmissible cancer Devil Facial Tumour Disease (DFTD), estimated to have originated between 1977 and 1987^44^, and causing a total population decline of 68% across 90% of their distribution by 2020^45^. Like other successful adapters, the devil has a wide habitat and dietary tolerance and few natural predators^46–51^, though their reliance on natural structures and soft soil for denning and shelter has prevented them from becoming entirely urbanised^9,52^. As specialised scavengers^53–55^, they are adept at exploiting food items provided by humans such as roadkill, dead livestock, and the carcasses of culled pest species^52,56,57^. However, pursuit of these resources and a preference for using artificial corridors such as roads for rapid movement through the landscape^58^ also puts them at great risk of road mortality^56^. Additionally, while certain prey items may be supplemented, others are likely removed through habitat destruction, reducing the diversity of available resources and the ability of individuals to reduce competition through dietary niche partitioning. This is of particular concern given that the most common period of DFTD transmission outside of the mating season is during communal feeding events^59^. As yet, the extent to which human activity influences the diet of devils, either positively or negatively, is unknown.

The Circular Head region in north-western Tasmania is a fitting location for testing such an effect as it contains habitat that forms a gradient of increasing anthropogenic impact. This gradient stretches from cleared pasture in the north, through wet eucalypt forest within permanent and future timber production zones, to the old-growth temperate rainforest of the northern Tarkine region^60^. These habitats differ greatly in the abundance of important devil food items like the Tasmanian pademelon (*Thylogale billardierii*), found in greater numbers in pasture than in dense native forests^61^. Pademelons and brushtail possums (*Trichosurus vulpecula*), another significant food item for devils, are also the species most commonly struck by vehicles in events that occur more frequently at high speeds (above 80 km/h) on highways^62^. However, the abundance of other potential food items can suffer for decades following the clearfelling and burning of production forests^63^. As humans influence the densities and activities of the species that devils feed on, so too do they likely affect the traits and behaviours of devils themselves.

The majority of research of devil diet has focused on using morphological scat and stomach content analysis of food items, indicating that, as a population, devils are generalist feeders that primarily consume medium to large mammals^47–49,51,64^. Both Guiler^47^ and Pemberton et al.^49^ show that there is variation in diet according to the resources available in different habitats, with birds more commonly found in coastal regions and introduced species near agricultural areas^47,49^. They were limited however, by their ability to only use short-term dietary data from scat or stomach contents containing food that had been consumed over approximately one day^65^. Bulk stable isotope analysis of nitrogen and carbon in whiskers has more recently been used for longer-term analysis of devil diets based on differences in age^66^, DFTD status^67^, and body size and intraspecific competition^54^ over several weeks or months. While nitrogen isotopic composition is frequently used to provide an indication of trophic position within the food web, as nutrients from food items are assimilated into the body tissue of consumers in a predictable manner^68–70^, carbon isotopic composition can differ significantly at the vegetation baseline of the ecosystem depending on the characteristics of plant species and environmental conditions^71–74^. By measuring both nitrogen and carbon isotopic ratios, the dietary niches of devils can be better determined and compared. However, this technique has yet to be used to investigate the effects of environmental conditions such as habitat on devil diet composition.

Here we analyse the diet of Tasmanian devils across a gradient of anthropogenic influence from rural agricultural land to undisturbed old-growth rainforest. Utilising bulk stable isotope analysis of nitrogen and carbon, we describe the trophic position and dietary niche and composition of devils in four habitats experiencing extensive to minor human impact. To determine whether there is evidence of dietary niche partitioning within the population, we then analyse the relationship between nitrogen and carbon stable isotope composition and individual characteristics including body size and sex.

## Methods

### Ethics statement

This study was conducted in accordance with the University of Sydney Animal Ethics Committee Permit Project 2017/1149, the NSW Animal Research Act 1985^75^, the Australian code for the care and use of animals for scientific purposes^76^, and ARRIVE guidelines^77^, with permission from the Tasmanian Department of Primary Industries, Parks, Water and Environment under scientific permit TFA 18149.

### Habitat classification

**Figure 1:**
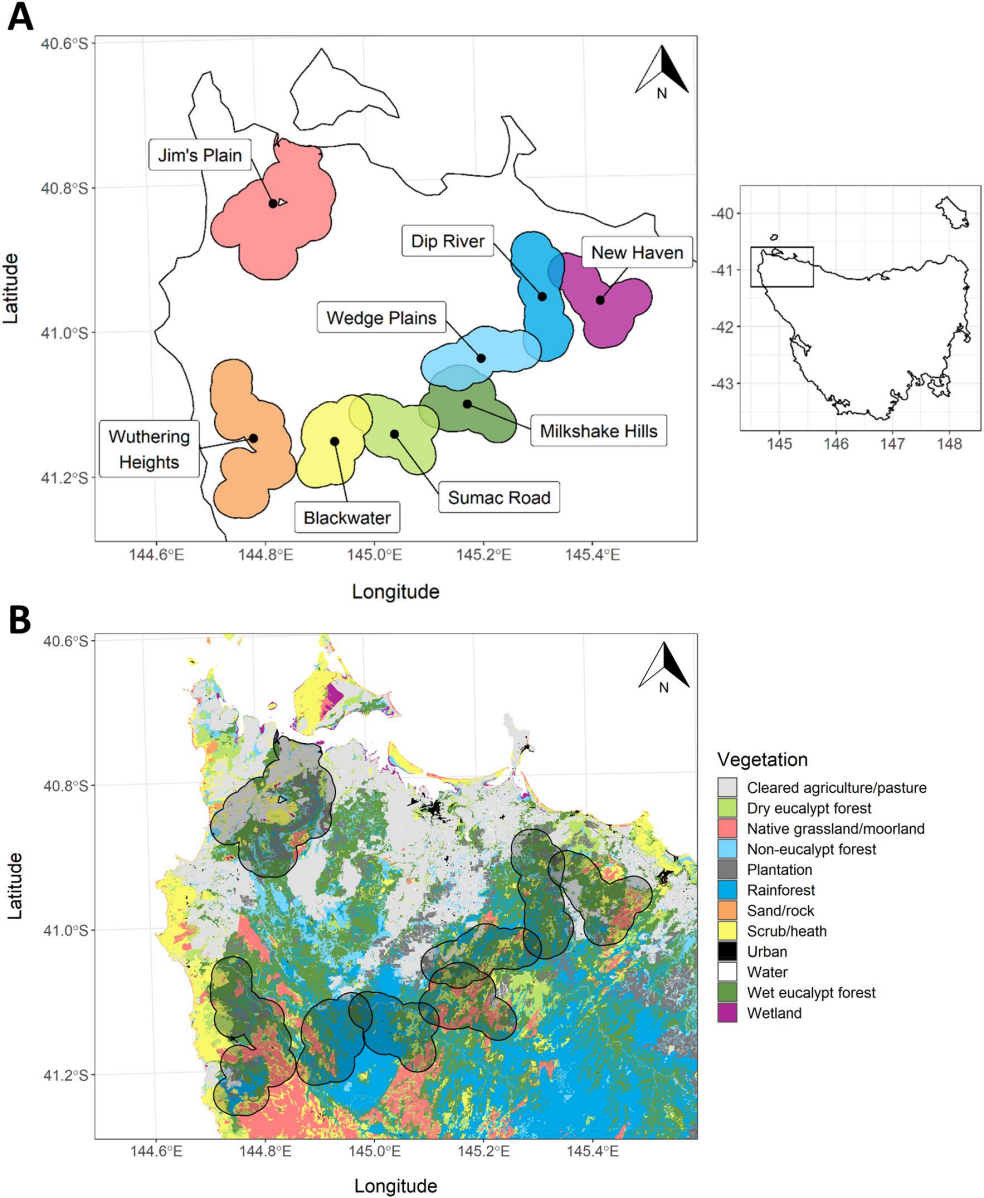
Map of north-west Tasmania, Australia generated using ArcGIS software^78^ and indicating (**A**) eight study sites and (**B**) 12 vegetation groups present within the Circular Head region based on data from TASVEG 4.0^79^.

Sampling occurred across eight study sites in the Circular Head region of north-west Tasmania (Fig. 1A), each containing 40 trapping locations. Surrounding the 320 trapping locations, we estimated a potential home range based on the average for the Tasmanian devil, 2,531 hectares^55^, and mapped each out using ArcGIS software^78^, defining the boundaries for each study site (Fig. 1A). Data from TASVEG 4.0^79^, a digital map of Tasmanian vegetation, were then overlayed, so that the dominant and any secondary habitat types surrounding each trapping location could be identified (Fig. 1B). TASVEG describes 82 mapping units in the Circular Head region, most of which are distinct vegetation communities. We classified these units into 12 broad groups, primarily based on those used by TASVEG, though with some differences (Supplementary Table 1).

**Figure 2:**
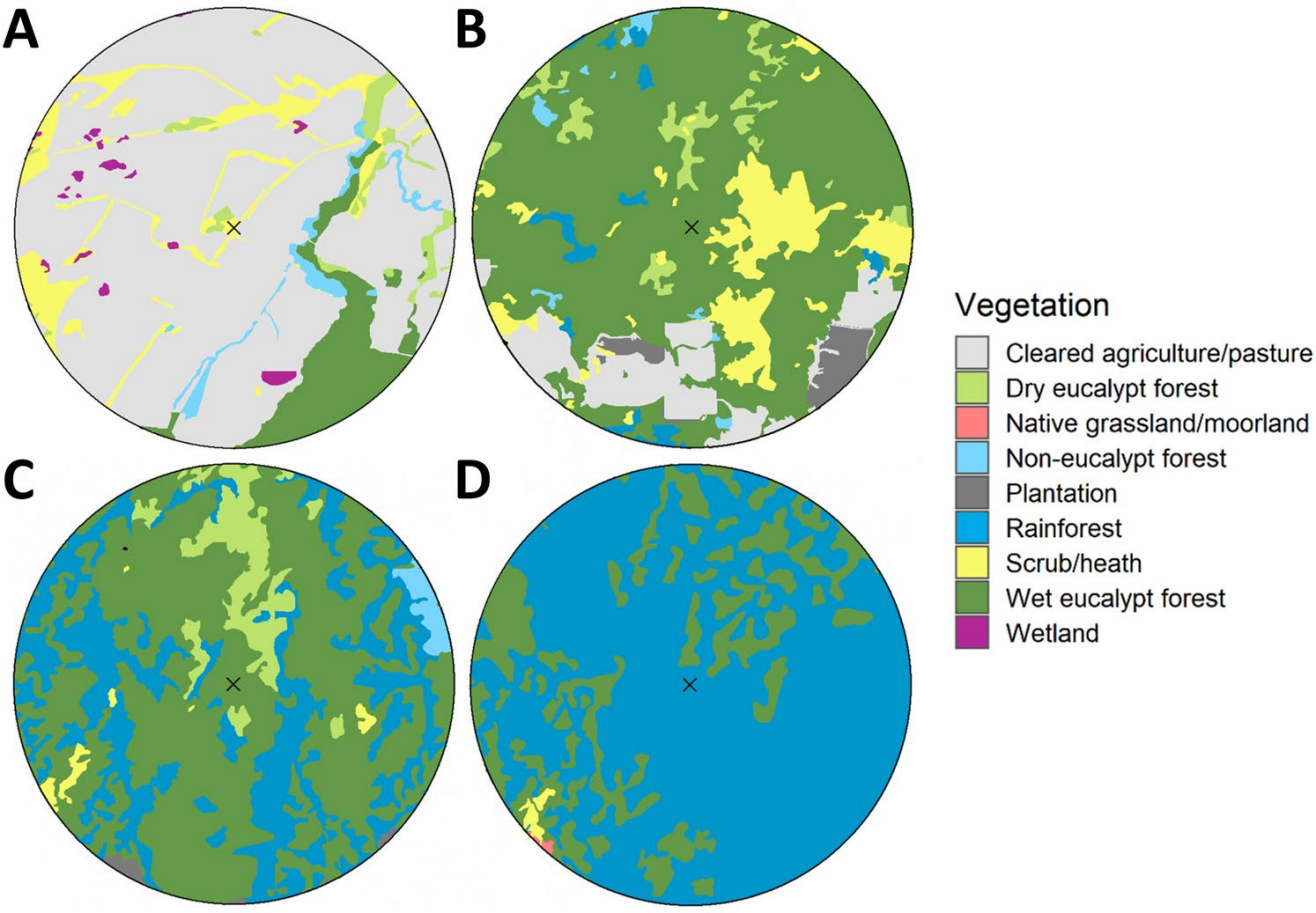
Examples of estimated 2,531 hectare home ranges surrounding four trapping locations (indicated by x) for each major habitat category: (**A**) cleared land; (**B**) wet eucalypt forest-other; (**C**) wet eucalypt forest-rainforest; and (**D**) rainforest.

The proportion of each estimated home range made up by these 12 vegetation groups was calculated and three dominant habitat types were identified: cleared land; rainforest; and wet eucalypt forest. The wet eucalypt forest category was further split by its secondary vegetation type: either rainforest or other (Fig. 2). Much of the wet eucalypt forest in the region is located within permanent timber production zones^80^ and has been harvested and regenerated over several decades^81^ on a rotation of 65–90 years^82^, while substantial regions of intact rainforest primarily grow within reserves^80^ and have been relatively undisturbed. Thus, these four vegetation categories form a gradient of anthropogenic impact from extensive (cleared land) to moderate (wet-eucalypt forest-other) to minor (wet-eucalypt forest-rainforest) to nil (rainforest).

### Whisker sample collection and preparation

118 individual devils were sampled between 26th June and 4th October 2018 and an additional seven between 25 and 30th June 2019, following overnight capture in custom-made PVC traps (N. Mooney and D. Ralph, unpublished data). Devils were selected randomly from a larger pool of individuals captured as part of an ongoing monitoring program. Each individual (n = 125; Supplementary Table 2) was prescribed a potential home range based upon the trap in which they were caught, with the assumption made that the trap was in the centre of the home range. Based on this potential home range, the devils were classified as feeding predominantly in one of the four habitat types: cleared land (n = 26); wet eucalypt forest-other (n = 43); wet eucalypt forest-rainforest (n = 22); or rainforest (n = 34). Their sex was recorded and age determined using standard indicators including canine eruption outlined for wild devils by Pemberton^83^. Individuals were categorised as either fully-grown adults (two to four years old) or yearlings (one year old) to account for differences in whisker growth patterns^84^. Juveniles under one year and older devils (five to six years old) were excluded due to low sample size, as the sampling period was outside of the juvenile dispersal season (December to March) and older devils do not persist in populations affected by DFTD. We used scissors to cut the longest posterior mystacial whisker of each individual as close as possible to the face and recorded its position within the whisker array^84^. As the length of the intradermal section of the whisker was unknown, an estimated length based on averages recorded by Attard et al. for each position was added to the measured extradermal length^84^. We then used a discrete von Bertalanffy equation^84–86^ to model the growth pattern of the whisker in order to cut lengths for stable isotope analysis that represented a few days. To remove lipids and other contaminants, whiskers were soaked for at least 20 min once in ultrapure water and twice in a 2:1 chloroform:methanol solution, then left to dry overnight. We measured and cut three segments of whisker weighing between 0.2 and 0.5 mg, ensuring a buffer section was left between each segment to allow for greater independence between samples. The three samples represented approximately 2.7 ± 1.5 days (mean ± sd) of growth each, which, when including the intervening buffer sections, covered about one month of isotope data altogether.

### Stable isotope analysis

Each whisker sample (n = 375) was placed into a tin capsule and combusted in an elemental analyser (Flash 2000 Organic Elemental Analyser, Thermo Scientific). A continuous flow isotope ratio mass spectrometer (Delta V Advantage, Thermo Scientific; Bioanalytical Mass Spectrometry Facility, University of New South Wales) was used to determine nitrogen (*δ*^15^N) and carbon (*δ*^13^C) isotope ratios using international standards USGS40 (*δ*^15^N_AIR_ = − 4.52 ± 0.06‰; *δ*^13^C_VPDB-LSVEC_ = − 26.39 ± 0.04‰) and USGS41a (*δ*^15^N_AIR_ = 47.55 ± 0.15‰; *δ*^13^C_VPDB-LSVEC_ = 36.55 ± 0.08‰)^87,88^ to correct for instrument drift and measurement error. Carbon isotope ratios are corrected to Vienna Pee Dee Belemnite (VPDB) and nitrogen isotope ratios to atmospheric N_2_ (Air)^89^. All isotope ratios are expressed as parts per thousand (‰) and were checked to ensure the carbon:nitrogen ratio fell between the recommended values of 2.9 and 3.8 for keratin^90^, with one sample with a ratio of 2.1 being removed. Two more samples fell below 2.9 (2.7 and 2.8), however as their corresponding nitrogen and carbon stable isotope values were very similar to those from the same individual, this appeared to be caused by reasonable variation and therefore they were not removed. Where instrument drift was too great for reliable correction (> 1.5‰), values were removed from analysis (nitrogen: 29 samples from 10 individuals; carbon: 25 samples from 9 individuals).

### Diet breadth analysis

Nitrogen and carbon isotopic niche sizes between devils in different habitats were calculated by using the SIBER package^91^ in R^92^ to fit Bayesian standard ellipse areas (SEA-B) with 95% confidence intervals.

The difference between nitrogen and carbon stable isotope values in each habitat was analysed using linear mixed models which included habitat, mass (as a proxy for body size), sex, and their interactions as fixed effects, and animal ID as a random effect. The R package MuMIn^93^ was used to find the model of best fit, selected based on the Akaike information criterion corrected for sample size (AICc). Models where ΔAICc < 2 are reported here as ones that are substantially supported^94^, and all tested models are included in Supplementary Tables 3 (*δ*^15^N) and 4 (*δ*^13^C). As the AICc values for *δ*^15^N were similar for all models where ΔAICc < 2, we chose the most parsimonious model by removing sex and fit a linear mixed model that included habitat, mass, and their interactions as fixed effects, and animal ID as a random effect. Tukey post-hoc pairwise comparisons were generated to analyse the overall difference in stable isotope values between habitats.

### Diet composition analysis

We analysed the diet composition of devils from different habitats using the Bayesian mixing model MixSIAR^95,96^ and rjags^97^ in R^92^ with an uninformative prior and the MCMC parameters: chain length = 2,000,000; burn = 1,000,000; thinning degree = 500; number of chains = 3. Raw *δ*^15^N and *δ*^13^C values for each individual devil were entered as consumer data, nested within habitat. Five likely prey groups collected from the same region and time period were chosen from Lewis et al.^54^ based on isotopic values and the raw values for each prey individual were added to the model. These groups were: (1) Tasmanian pademelon and European hare (*Lepus europaeus*); (2) brushtail possum; (3) red-necked wallaby (*Notamacropus rufogriseus*); (4) green rosella (*Platycercus caledonicus*); and (5) other birds (black currawong (*Strepera fuliginosa*); laughing kookaburra (*Dacelo novaeguineae*); and masked lapwing (*Vanellus miles*)). Discrimination factors from Newsome et al.^98^ were used to account for trophic enrichment. Results are reported as estimated proportions of total diet ± standard deviation.

## Results

### Isotopic niches

**Figure 3:**
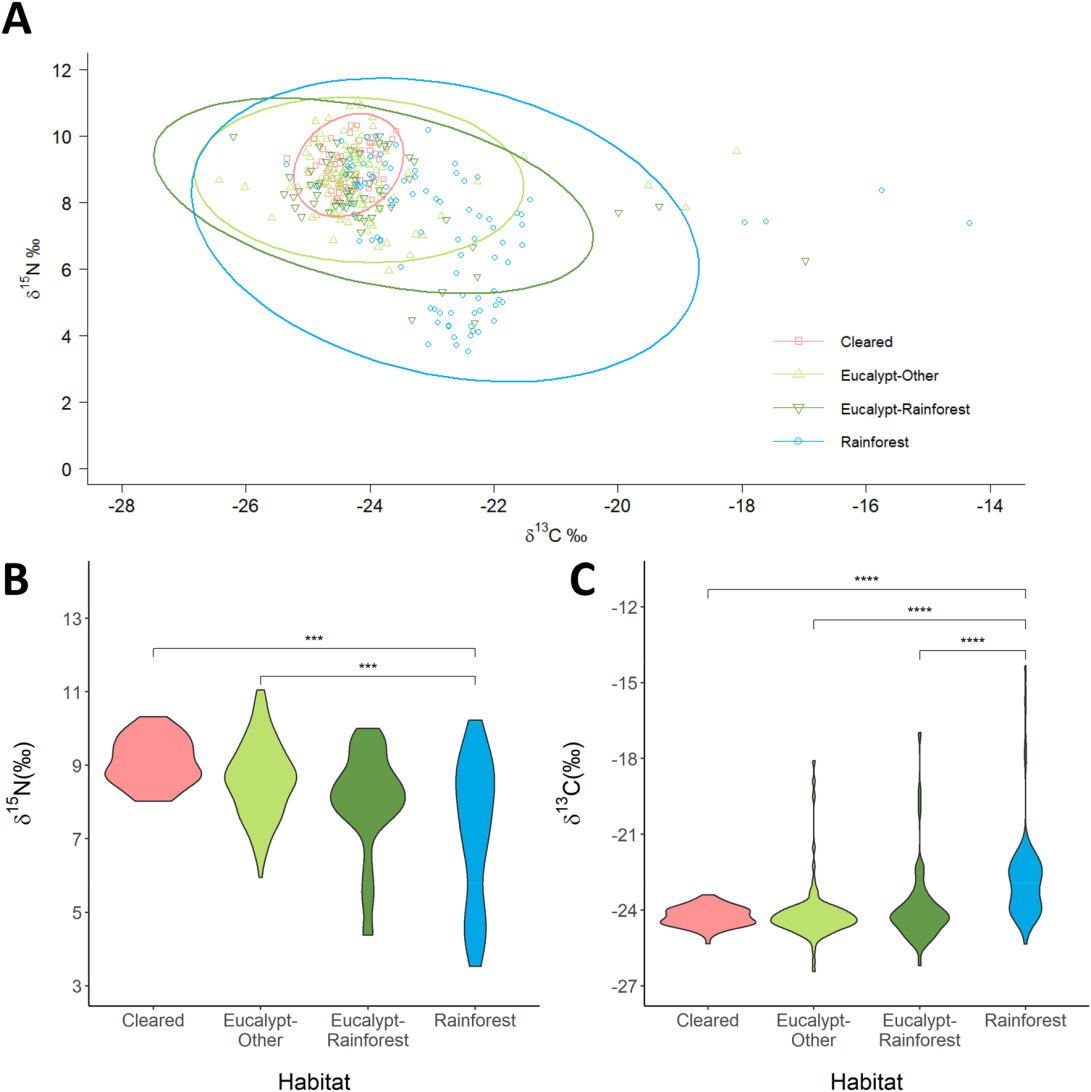
(**A**) Isotopic niches (CI = 0.95) for Tasmanian devils (n = 321 values from 109 individuals) from inhabiting four different habitats: cleared land (n = 19); wet eucalypt forest with other secondary habitat (n = 38); wet eucalypt forest with rainforest secondary habitat (n = 21); and rainforest (n = 31). (**B**) Violin plot of *δ*^15^N values (‰) for individuals (n = 116) in each habitat (cleared: n = 19; wet eucalypt-other: n = 43; wet eucalypt-rainforest: n = 22; rainforest: n = 32). (**C**) Violin plot of *δ*^13^C values (‰) for individuals (n = 118) in each habitat (cleared: n = 26; wet eucalypt-other: n = 38; wet eucalypt-rainforest: n = 21; rainforest: n = 33). Asterisks indicate *p*-values of Tukey post-hoc pairwise comparisons between habitats, where ****≤ 0.0001 and ***≤ 0.001.

Isotopic niches between habitats overlapped extensively without substantial separation. Devils inhabiting rainforest-dominated home ranges had the largest isotopic niche (SEA-B mode: 9.4; 95% CI 7.6–11.5; Fig. 3A) with *δ*^15^N values ranging from 3.5 to 10.2 (Fig. 3B) and *δ*^13^C values ranging from − 25.4 to − 14.3 (Fig. 3C). Those with home ranges dominated by cleared land had the smallest niche (SEA-B mode: 0.7; 95% CI 0.5–0.9; Fig. 3A), ranging from 8.0 to 10.3 for *δ*^15^N (Fig. 3B) and − 25.3 and − 23.4 for *δ*^13^C (Fig. 3C). In wet eucalypt forests, devils with home ranges where rainforest was the second-largest habitat type had a larger isotopic niche (SEA-B mode: 4.9; 95% CI 3.8–6.4; Fig. 3A) than those with a different secondary habitat (SEA-B mode: 3.4; 95% CI 2.8–4.1; Fig. 3A).

**Figure 4:**
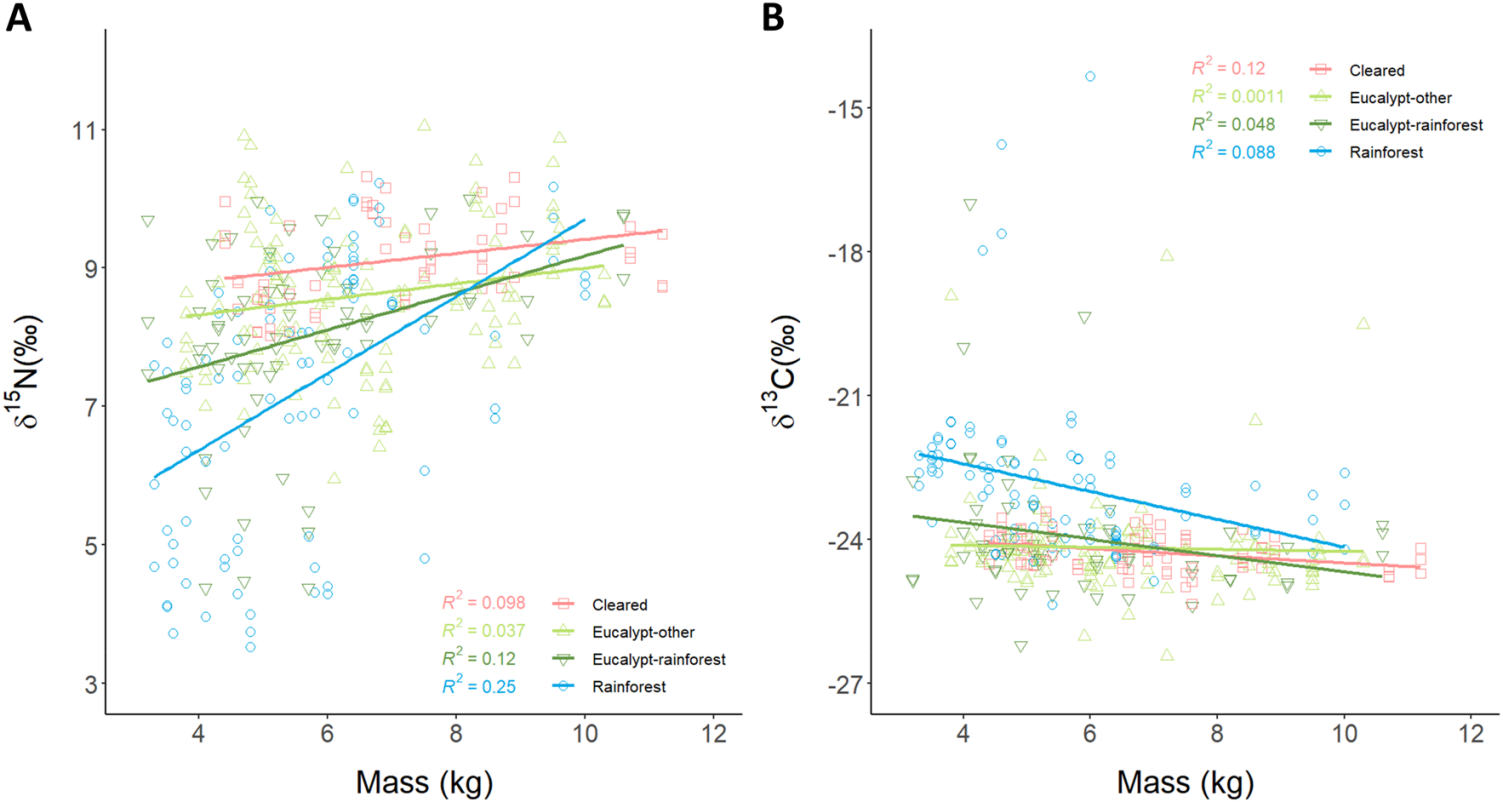
Relationships between (**A**) *δ*^15^N and (**B**) *δ*^13^C values (‰) and mass (kg) for Tasmanian devils (**A** n = 115; **B** n = 117). For *δ*^15^N values there is an interaction of habitat and mass, with only individuals in the rainforest showing a significant positive linear relationship between *δ*^15^N and mass (R^2^ = 0.25). There is a negative relationship between *δ*^13^C values and mass, with no interaction of habitat.

Habitat and mass were retained in all top-ranked model for *δ*^15^N (Table 1). Post-hoc Tukey pairwise comparisons of the most parsimonious model (after excluding sex) indicated that overall *δ*^15^N values were higher in cleared and wet eucalypt-other habitats than in rainforest ones (cleared—rainforest: t = 4.3, *p* < 0.001; wet eucalypt-other—rainforest: t = 3.8, *p* = 0.001; Fig. 3B). Heavier devils had higher *δ*^15^N values in rainforest habitats, but in other habitats there was no relationship between mass and *δ*^15^N (Fig. 4A).

**Table 1:** Summary of linear mixed models where ΔAIC_c_ < 2 for *δ*^15^N and *δ*^13^C values (‰).

Model rank	Intercept	Habitat	Mass	Sex	Habitat * Mass	Habitat * Sex	*df*	logLik	ΔAIC_c_	Weight	*p*
*δ*^15^N											
1	7.292	+	0.259				7	− 507.826	0.00	0.303	< 0.0001
2	8.402	+	0.101		+		10	− 505.110	0.90	0.193	< 0.0001
3	7.027	+	0.313	+		+	11	− 504.161	1.14	0.172	< 0.0001
4	7.011	+	0.310	+			8	− 507.544	1.53	0.141	< 0.0001
*δ*^13^C											
1	− 23.38	+	− 0.129				7	− 555.251	0.00	0.475	< 0.0001
2	− 24.25	+					6	− 557.131	1.68	0.206	< 0.0001

Habitat, sex, and mass (as a proxy for body size) were included as fixed variables, and animal ID as a random variable. *p* values are compared to the null model. All tested models are listed in Supplementary Tables 3 (*δ*^15^N) and 4 (*δ*^13^C).

The top-ranked model for *δ*^13^C values included habitat and mass (Table 1). Post-hoc Tukey pairwise comparisons indicated that overall *δ*^13^C values were higher in the rainforest than in all other habitats (cleared—rainforest: t = − 5.4, *p* < 0.0001; wet eucalypt-other—rainforest: t = − 5.8, *p* < 0.0001; wet eucalypt-rainforest—rainforest: t = − 4.4, *p* < 0.0001; Fig. 3C). Heavier devils had lower *δ*^13^C values overall (t = − 2.9, *p* < 0.01; Fig. 4B).

### Diet composition

**Figure 5:**
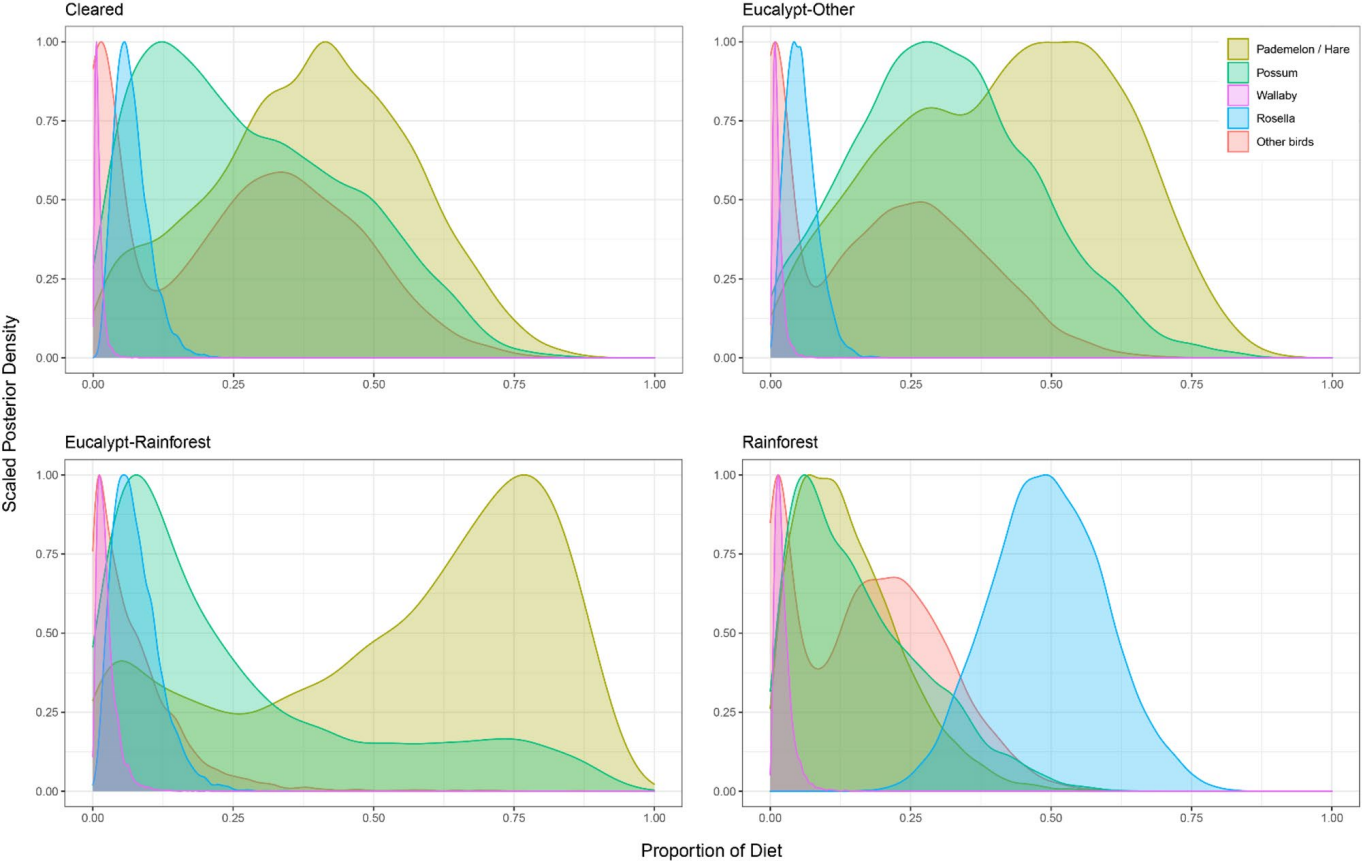
Posterior plot of the estimated diet composition of devils living in four habitats with differing anthropogenic impacts (cleared land, wet eucalypt-other, wet eucalypt-rainforest, rainforest). Five potential food groups were included in the mixing model based on Lewis et al.^54^: Tasmanian pademelon/European hare; brushtail possum; red-necked wallaby; green rosella; and other birds (black currawong; laughing kookaburra; and masked lapwing). Mean proportions, standard deviations and 95% CIs are included in Supplementary Table 5.

The diets of devils in cleared habitats were estimated to predominantly consist of the combined food group of Tasmanian pademelons and European hares (mean proportion ± sd: 0.39 ± 0.18), with brushtail possums making up 0.28 ± 0.18 and other birds an additional 0.26 ± 0.19 (Fig. 5). Pademelons/hares were also the most prominent food group for devils in wet eucalypt forests (wet eucalypt-other: 0.42 ± 0.20; wet eucalypt-rainforest: 0.55 ± 0.27) with possums estimated to be a secondary food source (wet eucalypt-other: 0.31 ± 0.16; wet eucalypt-rainforest: 0.27 ± 0.24) (Fig. 5). In rainforest habitats, green rosellas were estimated to be the primary food group making up 0.50 ± 0.10 of devil diet, with other birds forming 0.18 ± 0.13 and possums an additional 0.16 ± 0.12 (Fig. 5). All mean proportions, standard deviations, and 95% confidence intervals are summarised in Supplementary Table 5.

## Discussion

Tasmanian devil populations inhabiting old-growth rainforests had broader dietary niches than those in regenerated wet eucalypt forests and agricultural land, regions that have experienced greater anthropogenic disturbance. Those whose projected home ranges consisted primarily of cleared land had particularly restricted nitrogen and carbon isotope values, suggesting that individuals in these areas feed on similar food items. This provides evidence that habitat loss could have resulted in reduced biodiversity, a phenomenon commonly observed worldwide (as reviewed by Brooks et al.^99^, Fahrig^100^, and Pardini et al.^101^), resulting in fewer unique food items being available to devils. Individuals in wet eucalypt forests had similar stable isotope values to those in cleared areas, despite some individuals showing the potential to feed more broadly. A substantial proportion of these wet eucalypt forests are located in permanent timber production zones^80^ where the average rotation of harvesting falls between 65 and 90 years^82^. This is considerably shorter than the 140-year period required to form useful tree hollows for many native Tasmanian bird and mammal species^102^ and thus these forests likely lack the historical complexity of structure that may be supporting a greater species diversity in the relatively undisturbed rainforest. On the other hand, the narrower niches of devils, particularly in cleared regions, may indicate that anthropogenic influence has artificially inflated the availability of carcasses of particular species. This was observed in griffon vultures, where dietary niche breadths broadened following the closure of supplementary feeding stations^103^. Similarly, both deliberate and unintentional provision of carcasses to devils in rural areas through roadkill and hunting may make it unnecessary for individuals to seek out any other food items. Thus, it is possible that both reduced diversity and overpopulation of particular food items have worked in tandem to restrict the dietary niche of devils in both cleared and wet eucalypt habitats.

Rainforest-living devils had a comparatively broad isotopic niche and showed some evidence of partitioning, with heavier individuals having higher nitrogen stable isotope values than their smaller counterparts. Primary consumers in the Tasmanian isoscape do not all feed at the same nitrogen isotope ratio^54^. Instead, the higher nitrogen values found in heavier devils indicate that they were feeding primarily on medium-sized herbivorous mammals, while the lower values of smaller devils imply a diet of herbivorous birds^54^. This relationship was consistent with previous findings that better body condition was associated with higher nitrogen in DFTD-affected devils^67^ and carnivore body size being correlated with prey body size is a common trend observed between species^104–107^. Although species that frequently scavenge can get around the constraints of their body size, feeding on carcasses the same size or larger than themselves, greater competition surrounding these carcasses that are easier to find and harder to quickly remove still favours larger individuals^108^. In Tasmania, herbivorous mammal carcasses with higher nitrogen values, such as the Tasmanian pademelon, are the same or slightly smaller in size than the devil. It is therefore a cumbersome task for a smaller individual to attempt to remove a carcass to reduce competition from others. Also, although devils are often willing to feed at large carcasses with conspecifics, it is the larger adult males that are able to spend more time feeding undisturbed by others^109^. Thus, smaller devils may attempt to avoid competition for limited mammal carcasses by feeding more frequently on other smaller species. To investigate this further, stable isotope analysis should be used to investigate individual diets over longer periods of time (i.e., multiple seasons and years). The very low nitrogen values (< 6‰) observed here and at Bell et al. 2021’s West Pencil Pine site^67^ have only been found in devils with access to a substantial area of intact rainforest. This may indicate an under-sampling of these and other remote regions compared to those near human settlement. Thus, particular focus should be given to devils inhabiting rainforests, where there is greater diversity in diet, and native moorland and scrub, that have yet to be examined using stable isotope analysis.

The association between body size and nitrogen stable isotope values was not found among devils in the cleared and wet eucalypt habitats. Instead, almost all individuals fed within the same isotopic niche, potentially indicating a collapse of niche partitioning driven by human disturbance. This has also been observed between species^37–39^ and may result in increased competition for the same resources should they become limited. Most significantly, the majority of individuals within regenerated wet eucalypt forests had similar isotopic values to those in cleared land, though some fed more broadly, particularly where the secondary habitat within the estimated home range was rainforest. Although the potential artificial inflation of certain food items such as pademelon might reduce competition for food overall and support larger devil populations, it comes with the risk of increasing the number of social encounters that occur surrounding larger carcasses. This is of particular concern for a species threatened by two infectious cancers (DFTD1 and DFT2^110–112^) transmitted through biting behaviour^113^. Though transmission primarily occurs during antagonistic mating interactions in autumn (March to May), devils do also engage in fighting behaviour around carcasses found within their overlapping home ranges^59,109^. Having a more diverse diet that includes smaller food items may therefore reduce the risk of these encounters outside of the mating season.

Carbon stable isotope values were higher in the rainforest than in all other habitats. At first glance, our observations seem to contradict previous data, including within Tasmania^114^, where dryer ecosystems show higher carbon isotope ratios than closed-canopy, wet rainforests^115^. However, this provides further evidence that devils within the rainforest are feeding more frequently on birds (i.e., rosellas or other seed-feeding species such as cockatoos) that have travelled further afield than terrestrial-bound species are capable of. These birds are potentially feeding within dryer native moorland or scrub^114^, however, further investigation of the isotopic ratios within the region’s vegetation baseline is necessary to confirm this theory. Birds may be a particularly important dietary component for smaller individuals, who fed at higher carbon levels across all habitats, complementing previous observations made that juveniles and females feed more frequently on birds than adult males^57^.

Native moorland and dry eucalypt forest are notable devil habitats missing from our dataset due to their scarcity within our study sites, though within dry eucalypt-dominated habitat Bell et al. found similar isotope values to our devils in wet eucalypt forests^67^. Moorland shares similarities to cleared pasture in that it is an open habitat, yet it is unlikely to provide a high number of medium-sized mammal carcasses from anthropogenic sources such as roadkill or hunting. To confirm whether dietary limitation is driven primarily by anthropogenic supplementation of food items or the openness of the habitat, a similar analysis of diet in moorland should be conducted. Nonetheless, human activity in the region (e.g., forestry) contributes to both potential drivers by reducing vegetation density, and therefore ultimately would retain some role in any resulting decrease in dietary diversity.

Tasmanian pademelons and European hares were predicted to make up the largest proportion of diet for devils living in the cleared and regenerated wet eucalypt forest habitats, matching the preference of those species for feeding in open pasture and mosaic landscapes rather than in dense native forest^61,116^. This also complements research that utilised morphological scat and stomach analysis to find that medium sized mammals made up a substantial proportion of devil diets^47–49,51,64^. Although we were unable to split pademelons and hares from each other due to their heavily overlapping isotope signatures, it can be assumed this group was predominantly represented by the pademelon, as their previously reported abundance as roadkill was over 50 times that of hares^62^. Another source of food within this isotopic group of grass-feeding, medium-sized mammals may be the European rabbit (*Oryctolagus cuniculus*).

Conversely, it was estimated that green rosellas form a substantial proportion of rainforest devil diet, potentially indicating their greater abundance or the reduced availability of larger mammal carcasses. Though our mixing model only included green rosellas due to the availability of prey sample stable isotope values from the region, it can be assumed that other primarily seed-feeding rainforest-inhabiting bird species (e.g., yellow-tailed black cockatoos, *Calyptorhynchus funereus*^117^) could belong to the same functional group. On top of dense old-growth rainforest providing greater cover for nesting, these birds may behave differently within the perceived greater safety of a closed forest compared with more open and therefore potentially more dangerous landscapes. In temperate rainforests, green rosellas spend more time within the canopy^118^, making them a challenging target for the devil, better known for being a skilled scavenger than a hunter or climber. However, a substantial proportion of animal deaths will occur due to reasons other than direct predation^119^, including starvation, disease, injury, and exposure. In a system with few predators such as the Tasmanian temperate rainforest, the rate of “natural” deaths of bird species may be great enough to sustain a devil population alongside the more occasional availability of larger mammalian carcasses. Alternatively, devils may feed on these birds at roadsides and ecotones with less tree cover, where they are more likely to spend time on the ground and be struck by vehicles.

We found the model estimated a low reliance on the red-necked wallaby, the largest included food item, within all habitats, despite previous observations of the species being an important food source for devils^47–49,51^. This may be explained in part by local differences in wallaby and pademelon abundance, as densities of the two species are similar in south-east and central regions, while pademelons are comparatively more abundant in the north and south-west^120^. Larger mammals are also less likely to be killed on the roads compared to pademelons or brushtail possums^62,121^. However, caution should be exercised when interpreting mixing model results here, as our analysis uses the default uninformative prior^95^ and focuses on a small number of food item sources that are predicted to form the largest proportion of their diet. For this reason, we have only discussed the food groups estimated to make up the largest proportion of the diet of each devil population. Devils are capable of feeding broadly^46–49,51,64^ and there is a lot of isotopic overlap between potential food items. Stable isotope values for food items within the region were also collected opportunistically and it is likely that many species consumed by devils are missing from this analysis. Additionally, we made assumptions that each trapping location was centred within an individual’s estimated home range, that each home range was the same size, and that individuals foraged throughout their estimated home range, though in reality this is unlikely. Precise observations of a devil’s diet, habitat use during foraging, and home range size can be recorded using radio tracking or the use of GPS and video collars^55,58,83^, however time and financial constraints require that these techniques be limited to relatively few individuals. These assumptions, along with the use of stable isotope analysis, allowed us to maximise sample size instead. Thus, this study is a starting point that in the future could be compared with other techniques to analyse the dietary niche and composition of devils between habitats.

We found that the dietary niches of Tasmanian devil populations become more restricted along a habitat gradient from dense, undisturbed temperate rainforest to highly human-modified cleared land. Devils inhabiting cleared land and regenerated native forest likely benefit from the artificial inflation and reliable supply of some food items. However, a lack of diversity in their diets as a population could indicate the absence of certain other species and the collapse of niche partitioning that may have served as a vital tool to reduce aggressive interactions around food. Of particular note is our observation that the dietary niches of devil populations in regenerated wet eucalypt forests more closely resembled those in cleared land than in old-growth rainforest, indicating the conservation value of the latter for the devil and the species which they consume.

## Supplementary Information

Supplementary Information 1.

Supplementary Information 2.

## Data Availability

All data generated or analysed during this study is included within the published article and its supplementary information.
