# Supplementary material for: Living in human-modified landscapes narrows the dietary niche of a specialised mammalian scavenger
Source: Sci Rep. 2023 Mar 3;13:3582. doi: 10.1038/s41598-023-30490-6 (PMC9984462; doi:10.1038/s41598-023-30490-6)
Supplement: Supplementary file 1 — Supplementary Information 1. [file 41598_2023_30490_MOESM1_ESM.docx]

**Supplementary Table 1.**The classification of 82 mapping units identified in the TASVEG mapping dataset for the Circular Head region of north-west Tasmania, Australia into 12 vegetation groups.

| **Vegetation Group** | **TASVEG Code** | **TASVEG Mapping Unit** |
| --- | --- | --- |
| **Cleared agricultural land and pasture** | FAC | Improved pasture with native tree canopy |
|  | FAG | Agricultural land |
|  | FMG | Marram grassland |
|  | FPF | *Pteridum esculentum*fernland |
|  | FRG | Regenerating cleared land |
|  | FWU | Weed infestation |
| **Dry eucalypt forest**  **and woodland** | DAC | *Eucalyptus amygdalina*coastal forest and woodland |
|  | DNI | *Eucalyptus nitida*dry forest and woodland |
|  | DOB | *Eucalyptus obliqua*dry forest |
|  | DOV | *Eucalyptus ovata*forest and woodland |
|  | DVC | *Eucalyptus viminalis-Eucalyptus globulus*coastal forest and woodland |
|  | DVG | *Eucalyptus viminalis*grassy forest and woodland |
| **Native grassland  and moorland** | GHC | Coastal grass and herbfield |
|  | GPH | Highland *Poa* grassland |
|  | GPL | Lowland *Poa labillardierei*grassland |
|  | GSL | Lowland grassy sedgeland |
|  | GTL | Lowland *Themeda triandra*grassland |
|  | MBE | Eastern buttongrass moorland |
|  | MBP | Pure buttongrass moorland |
|  | MBR | Sparse buttongrass moorland on slopes |
|  | MBS | Buttongrass moorland with emergent shrubs |
|  | MBU | Undifferentiated buttongrass moorland |
|  | MBW | Western buttongrass moorland |
|  | MRR | Restionaceae rushland |
|  | MSW | Western lowland sedgeland |
| **Non-eucalypt forest and woodland** | NAD | *Acacia dealbata*forest |
|  | NAF | *Acacia melanoxylon*swamp forest |
|  | NAR | *Acacia melanoxylon*forest on rises |
|  | NAV | *Allocasuarina verticillata*forest |
|  | NBS | *Banksia serrata*woodland |
|  | NLE | *Leptospermum*forest |
|  | NLM | *Leptospermum lanigerum-Melaleuca squarrosa*swamp forest |
|  | NME | *Melaleuca ericifolia*swamp forest |
| **Plantation for silviculture** | FPH | Hardwood plantations for silviculture |
|  | FPS | Softwood plantations for silviculture |
|  | FPU | Unverified plantations for silviculture |
| **Vegetation Group** | **TASVEG Code** | **TASVEG Mapping Unit** |
| **Rainforest and related scrub** | RHP | *Lagarostrobos franklinii*rainforest and scrub |
|  | RML | *Nothofagus-Leptospermum*short rainforest |
|  | RMS | *Nothofagus-Phyllocladus*short rainforest |
|  | RMT | *Nothofagus-Atherosperma*rainforest |
|  | RMU | Undifferentiated *Nothofagus*rainforest |
| **Sand and rock** | ORO | Lichen lithosere |
|  | OSM | Sand, mud |
| **Scrub, heathland**  **and coastal complexes** | SAL | *Acacia longifolia*coastal scrub |
|  | SBR | Broad-leaf scrub |
|  | SCA | Coastal scrub on alkaline sands |
|  | SCH | Coastal heathland |
|  | SHS | Subalpine heathland |
|  | SHW | Wet heathland |
|  | SLG | *Leptospermum glaucescens*heathland and scrub |
|  | SLL | *Leptospermum lanigerum*scrub |
|  | SLS | *Leptospermum scoparium*heathland and scrub |
|  | SMM | *Melaleuca squamea*heathland |
|  | SMR | *Melaleuca squarrosa*scrub |
|  | SRF | *Leptospermum*with rainforest scrub |
|  | SRH | Rookery halophytic herbland |
|  | SSC | Coastal scrub |
|  | SSW | Western subalpine scrub |
|  | SSZ | Spray zone coastal complex |
|  | SWR | Western regrowth complex |
|  | SWW | Western wet scrub |
| **Urban areas and infrastructure** | FPE | Permanent easements |
|  | FUM | Extra-urban miscellaneous |
|  | FUR | Urban areas |
| **Water** | OAQ | Water, sea |
| **Wet eucalypt forest and woodland** | WBR | *Eucalyptus brookeriana*wet forest |
|  | WDU | Undifferentiated *Eucalyptus delegatensis*wet forest |
|  | WNL | *Eucalyptus nitida*forest over *Leptospermum* |
|  | WNR | *Eucalyptus nitida*forest over rainforest |
|  | WNU | Undifferentiated *Eucalyptus nitida*wet forest |
|  | WOB | *Eucalyptus obliqua*forest with broad-leaf shrubs |
|  | WOL | *Eucalyptus obliqua*forest over *Leptospermum* |
|  | WOR | *Eucalyptus obliqua*forest over rainforest |
|  | WOU | Undifferentiated *Eucalyptus obliqua*wet forest |

| **Vegetation Group** | **TASVEG Code** | **TASVEG Mapping Unit** |
| --- | --- | --- |
| **Wetland and saltmarsh** | AHF | Freshwater aquatic herbland |
|  | AHL | Lacustrine herbland |
|  | AHS | Saline aquatic herbland |
|  | ARS | Saline sedgeland/rushland |
|  | ASF | Freshwater aquatic sedgeland and rushland |
|  | ASS | Succulent saline herbland |
|  | AUS | Undifferentiated saltmarsh |
|  | AWU | Undifferentiated wetland |

**Supplementary Table 3.** Summary of linear mixed models for *δ*^15^N values (‰). Habitat, sex, mass (as a proxy for body size), and their interactions were included as fixed variables, and animal ID as a random variable. *p* values are compared to the null model.

|  | ***δ*^15^N** | | | | | | | | | | | | | |
| --- | --- | --- | --- | --- | --- | --- | --- | --- | --- | --- | --- | --- | --- | --- |
| **Model rank** | | **Intercept** | **Habitat** | **Mass** | **Sex** | **Habitat*Mass** | **Habitat*Sex** | **Mass*Sex** | **Habitat*Mass*Sex** | ***df*** | **logLik** | **ΔAIC_c_** | **Weight** | ***p*** |
| 1 | | 7.292 | + | 0.259 |  |  |  |  |  | 7 | -507.826 | 0.00 | 0.303 | 0.00000000010 |
| 2 | | 8.402 | + | 0.101 |  | + |  |  |  | 10 | -505.110 | 0.90 | 0.193 | 0.00000000002 |
| 3 | | 7.027 | + | 0.313 | + |  | + |  |  | 11 | -504.161 | 1.14 | 0.172 | 0.00000000071 |
| 4 | | 7.011 | + | 0.310 | + |  |  |  |  | 8 | -507.544 | 1.53 | 0.141 | 0.00000000021 |
| 5 | | 8.165 | + | 0.147 | + | + |  |  |  | 11 | -504.813 | 2.44 | 0.090 | 0.00000000003 |
| 6 | | 8.585 | + | 0.066 | + | + | + |  |  | 14 | -502.684 | 4.67 | 0.029 | 0.00000000040 |
| 7 | | 6.841 | + | 0.343 | + |  | + | + |  | 12 | -505.071 | 5.10 | 0.024 | 0.00000000195 |
| 8 | | 6.667 | + | 0.369 | + |  |  | + |  | 9 | -508.381 | 5.32 | 0.021 | 0.00000000061 |
| 9 | | 8.114 | + | 0.155 | + | + |  | + |  | 12 | -505.856 | 6.67 | 0.011 | 0.00000000009 |
| 10 | | 7.794 | + | 0.192 | + | + | + | + | + | 18 | -499.399 | 6.93 | 0.009 | 0.00000000030 |
| 11 | | 8.379 | + | 0.099 | + | + | + | + |  | 15 | -503.395 | 8.28 | 0.005 | 0.00000000086 |
| 12 | | 9.116 | + |  |  |  |  |  |  | 6 | -514.864 | 11.99 | 0.001 | 0.00000019540 |
| 13 | | 9.022 | + |  | + |  | + |  |  | 7 | -514.204 | 12.76 | 0.001 | 0.00000093990 |
| 14 | | 9.004 | + |  | + |  |  |  |  | 10 | -511.046 | 12.77 | 0.001 | 0.00000022170 |
| 15 | | 5.844 |  | 0.416 | + |  |  |  |  | 5 | -517.679 | 15.55 | 0.000 | 0.00000018900 |
| 16 | | 6.086 |  | 0.342 |  |  |  |  |  | 4 | -518.998 | 16.13 | 0.000 | 0.00000015620 |
| 17 | | 5.232 |  | 0.531 | + |  |  | + |  | 6 | -517.917 | 18.10 | 0.000 | 0.00000041570 |
| 18 | | 8.194 |  |  |  |  |  |  |  | 3 | -530.769 | 37.62 | 0.000 |  |
| 19 | | 8.050 |  |  | + |  |  |  |  | 4 | -530.348 | 38.83 | 0.000 | 0.18780000000 |
|  |  | | | | | | | | | | | | | |

**Supplementary Table 4.** Summary of linear mixed models for *δ*^13^C values (‰). Habitat, sex, mass (as a proxy for body size), and their interactions were included as fixed variables, and animal ID as a random variable. *p* values are compared to the null model.

|  | ***δ*^13^C** | | | | | | | | | | | | | |
| --- | --- | --- | --- | --- | --- | --- | --- | --- | --- | --- | --- | --- | --- | --- |
| **Model rank** | | **Intercept** | **Habitat** | **Mass** | **Sex** | **Habitat*Mass** | **Habitat*Sex** | **Mass*Sex** | **Habitat*Mass*Sex** | ***df*** | **logLik** | **ΔAIC_c_** | **Weight** | ***p*** |
| 1 | | -23.38 | + | -0.129 |  |  |  |  |  | 7 | -555.251 | 0.00 | 0.475 | 0.00000000005 |
| 2 | | -24.25 | + |  |  |  |  |  |  | 6 | -557.131 | 1.68 | 0.206 | 0.00000000066 |
| 3 | | -24.16 | + |  | + |  |  |  |  | 7 | -556.364 | 2.23 | 0.156 | 0.00000000056 |
| 4 | | -23.41 | + | -0.123 | + |  |  |  |  | 8 | -555.917 | 3.43 | 0.086 | 0.00000000019 |
| 5 | | -24.20 | + |  | + |  | + |  |  | 10 | -554.708 | 5.24 | 0.035 | 0.00000000539 |
| 6 | | -23.44 | + | -0.124 | + |  | + |  |  | 11 | -554.223 | 6.40 | 0.019 | 0.00000000145 |
| 7 | | -23.10 | + | -0.176 | + |  |  | + |  | 9 | -556.952 | 7.61 | 0.011 | 0.00000000053 |
| 8 | | -23.75 | + | -0.074 |  | + |  |  |  | 10 | -556.114 | 8.05 | 0.008 | 0.00000000013 |
| 9 | | -23.21 | + | -0.162 | + |  | + | + |  | 12 | -555.328 | 10.76 | 0.002 | 0.00000000366 |
| 10 | | -23.77 | + | -0.069 | + | + |  |  |  | 11 | -556.799 | 11.56 | 0.001 | 0.00000000040 |
| 11 | | -23.76 | + | -0.071 | + | + | + |  |  | 14 | -555.175 | 14.79 | 0.000 | 0.00000000350 |
| 12 | | -23.61 | + | -0.098 | + | + |  | + |  | 12 | -558.007 | 16.12 | 0.000 | 0.00000000106 |
| 13 | | -23.54 | + | -0.108 | + | + | + | + |  | 15 | -556.070 | 18.76 | 0.000 | 0.00000000676 |
| 14 | | -23.63 | + | -0.093 | + | + | + | + | + | 18 | -555.243 | 23.75 | 0.000 | 0.00000002330 |
| 15 | | -22.55 |  | -0.201 |  |  |  |  |  | 4 | -572.223 | 27.73 | 0.000 | 0.00008315000 |
| 16 | | -22.51 |  | -0.212 | + |  |  |  |  | 5 | -572.750 | 30.84 | 0.000 | 0.00040830000 |
| 17 | | -21.92 |  | -0.324 | + |  |  | + |  | 6 | -572.865 | 33.14 | 0.000 | 0.00048380000 |
| 18 | | -23.78 |  |  |  |  |  |  |  | 3 | -577.814 | 36.86 | 0.000 |  |
| 19 | | -23.63 |  |  | + |  |  |  |  | 4 | -576.899 | 37.08 | 0.000 | 0.07003000000 |

**Supplementary Table 5.**Summary statistics for MixSIAR output indicating the estimated mean proportion of Tasmanian devil diet formed by five potential food groups in four habitats.

| **Habitat** | **Food group** | **Mean** | **σ** | **95% CI** |
| --- | --- | --- | --- | --- |
| **Cleared** | *Pademelon-Hare* | 0.39 | 0.18 | 0.07 - 0.67 |
|  | *Possum* | 0.28 | 0.18 | 0.04 - 0.60 |
|  | *Wallaby* | 0.01 | 0.01 | 0.00 - 0.02 |
|  | *Rosella* | 0.07 | 0.03 | 0.03 - 0.13 |
|  | *Other birds* | 0.26 | 0.19 | 0.00 - 0.56 |
| **Eucalypt-Other** | *Pademelon-Hare* | 0.42 | 0.20 | 0.09 - 0.72 |
|  | *Possum* | 0.31 | 0.16 | 0.06 - 0.60 |
|  | *Wallaby* | 0.01 | 0.01 | 0.00 - 0.03 |
|  | *Rosella* | 0.06 | 0.03 | 0.02 - 0.11 |
|  | *Other birds* | 0.20 | 0.16 | 0.00 - 0.47 |
| **Eucalypt-Rainforest** | *Pademelon-Hare* | 0.55 | 0.27 | 0.04 - 0.87 |
|  | *Possum* | 0.27 | 0.24 | 0.02 - 0.79 |
|  | *Wallaby* | 0.03 | 0.02 | 0.01 - 0.06 |
|  | *Rosella* | 0.08 | 0.04 | 0.02 - 0.16 |
|  | *Other birds* | 0.08 | 0.09 | 0.00 - 0.24 |
| **Rainforest** | *Pademelon-Hare* | 0.14 | 0.10 | 0.02 - 0.32 |
|  | *Possum* | 0.16 | 0.12 | 0.02 - 0.39 |
|  | *Wallaby* | 0.02 | 0.02 | 0.01 - 0.05 |
|  | *Rosella* | 0.50 | 0.13 | 0.25 – 0.66 |
|  | *Other birds* | 0.18 | 0.13 | 0.00 - 0.39 |
